# Cardamonin, a Novel Antagonist of hTRPA1 Cation Channel, Reveals Therapeutic Mechanism of Pathological Pain

**DOI:** 10.3390/molecules21091145

**Published:** 2016-08-29

**Authors:** Shifeng Wang, Chenxi Zhai, Yanling Zhang, Yangyang Yu, Yuxin Zhang, Lianghui Ma, Shiyou Li, Yanjiang Qiao

**Affiliations:** 1 Key Laboratory of TCM-Information Engineer of State Administration of TCM, School of Chinese Materia Medica, Beijing University of Chinese Medicine, No. 6 Wangjing Zhonghuan South Road, Chaoyang District, Beijing 100102, China; wangshifeng@bucm.edu.cn (S.W.); zhaichenxi1991@163.com (C.Z.); collean_zhang@163.com (Y.Z.); louisyang@bucm.edu.cn (Y.Y.); zhangyuxinwjzy@163.com (Y.Z.); 2 HD Biosciences, Co., Ltd., 590 Ruiqing Road, Zhangjiang Hi-Tech Park East Campus, Pudong New Area, Shanghai 201201, China; malianghui@hdbiosciences.com; 3 Key Laboratory of Genomic and Precision Medicine, Beijing Institute of Genomics, Chinese Academy of Sciences, No. 1 Beichen West Road, Chaoyang District, Beijing 100101, China

**Keywords:** *Alpinia katsumadai hayata*, cardamonin, TRPA1 antagonist, hyperalgeisa, cardiotoxicity

## Abstract

The increasing demand for safe and effective treatments of chronic pain has promoted the investigation of novel analgesic drugs. Some herbals have been known to be able to relieve pain, while the chemical basis and target involved in this process remained to be clarified. The current study aimed to find anti-nociceptive candidates targeting transient receptor potential ankyrin 1 (TRPA1), a receptor that implicates in hyperalgesia and neurogenic inflammation. In the current study, 156 chemicals were tested for blocking HEK293/TRPA1 ion channel by calcium-influx assay. Docking study was conducted to predict the binding modes of hit compound with TRPA1 using Discovery Studio. Cytotoxicity in HEK293 was conducted by Cell Titer-Glo assay. Additionally, cardiotoxicity was assessed via xCELLigence RTCA system. We uncovered that cardamonin selectively blocked TRPA1 activation while did not interact with TRPV1 nor TRPV4 channel. A concentration-dependent inhibitory effect was observed with IC_50_ of 454 nM. Docking analysis of cardamonin demonstrated a compatible interaction with A-967079-binding site of TRPA1. Meanwhile, cardamonin did not significantly reduce HEK293 cell viability, nor did it impair cardiomyocyte constriction. Our data suggest that cardamonin is a selective TRPA1 antagonist, providing novel insight into the target of its anti-nociceptive activity.

## 1. Introduction

*Alpinia katsumadai hayata* (*A. katsumadai*) belongs to pungent and warm herbal medicine in the classification of traditional Chinese medicines, which possesses effects of warming the stomach, removing damp-cold in the spleen, and arresting vomiting. It is, thus, applied for gastric pain and distended abdomen. Neuro-protection effect of *A. katsumadai* for ischemic damage has been observed through antioxidants [1,2]. Choi et al. [3] showed that extracts of *A. katsumadai* attenuate phenylbenzoquinone-induced writhing and carrageenan-induced hyperalgesia, and they found that this effect was independent of opioid receptor but involved in COX-2 pathway, suggesting potential role of *A. katsumadai* on inflammatory nociception. Cardamonin (2, 4-dihydroxy-6-methoxychalcone), a major component isolated from the seed of cardamom spices, occupies approximately 0.5% in *A. katsumadai* [4]. According to a study of Park et al. [5], cardamonin was a major active ingredient of *A. katsumadai* that was responsible for the anti-nociceptive effect. Reduced inflammatory factors, such as cycloosygenase-2 (COX-2) and transglutaminase-2 (Tgase-2), were involved in the anti-nociceptive action. Nonetheless, the molecular target of cardamonin concerning analgesic efficacy still remained unknown.

Transient receptor potential channels (TRP) are involved in activation or modulation of pain signal transduction pathways [6,7]. Transient receptor potential ankyrin 1 (TRPA1) is an intrinsically cold-activated and chemo sensitive ion channel [8], which is also known as a wasabi receptor [9,10]. It is originally named as ankyrin-like with transmembrane domains protein 1 (ANKTM1) [11]. TRPA1 is conserved in many species during evolution and is largely expressed in sensory neurons [12,13]. Recognized roles of TRPA1 included as a mediator of somatosensory processes and nociceptive transmission. It has been observed that TRPA1 is over-activated by inflammatory mediators, such as prostaglandins and fatty acid metabolites [14], thus leading to hyperalgeisa [15].

Considering that antagonists of the TRPA1 cation channel present as a promising group of drugs for controlling persistent pain [16], the current study aimed to identify novel a TRPA1 antagonist from traditional Chinese medicines.

## 2. Results

### 2.1. Identification of Cardamonin as a Novel TRPA1 Antagonist

**Figure 1:**
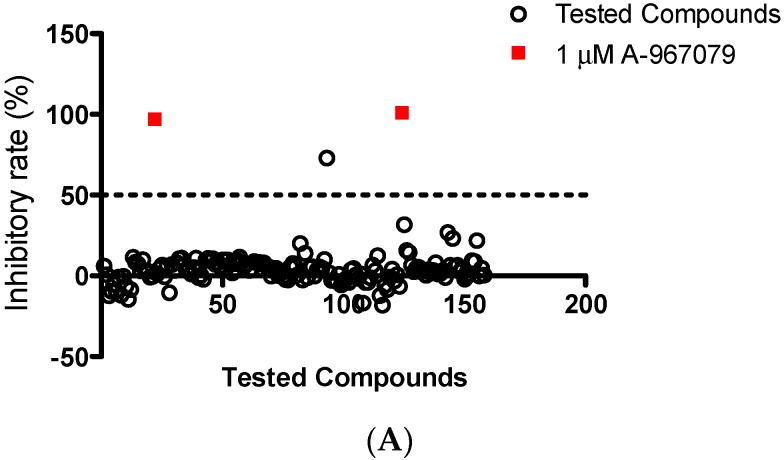

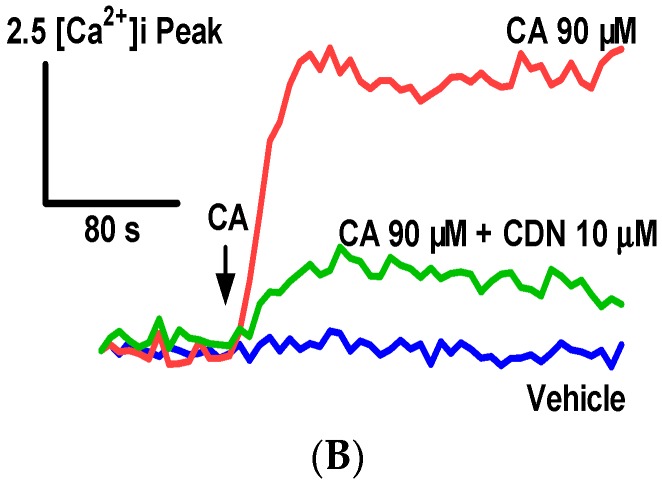
Identification of cardamonin (CDN) as a TRPA1 antagonist. (**A**) Scatterplot of inhibitory rates of 156 herbal derived chemicals on TRPA1 activation. Cinnamaldehyde (CA) was applied as a TRPA1 agonist to stimulate calcium mobilization at 90 μM. A-967079 (1 μM) was used as a positive TRPA1 antagonist control. 50% inhibition rate was defined as the cutoff; and (**B**) representative time-course profile of CDN on TRPA1 agonist CA stimulated calcium signal. The arrow indicates the onset of 90 μM CA.

To discover a novel TRPA1 antagonist, a small library containing 156 natural chemicals were evaluated for their inhibitory effects on the TRPA1 ion channel at 10 μM (Figure 1A). The compound list was released previously [17]. The cells were pre-incubated with cinnamaldehyde (CA) by its EC_80_ (90 μΜ) [18,19] for 10 min and then treated with tested compounds. The assay identified one hit, cardamonin (10 μM), which significantly inhibited CA induced HEK293/TRPA1 activation. The time-course profile of the calcium signal was recorded (Figure 1B) and the peak value was used for determination of the maximum calcium mobilization.

**Figure 2:**
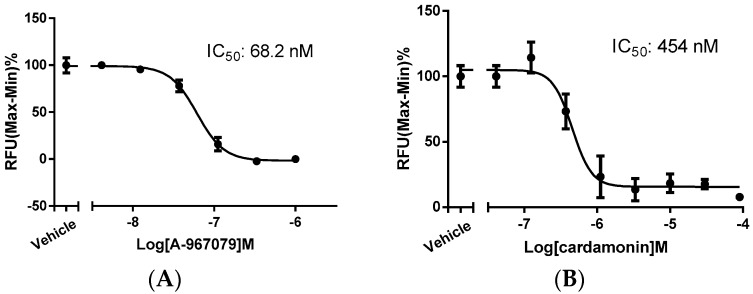
Antagonistic concentration-response determination of A-967079 and cardamonin. (**A**) Concentration-response curve for A-967079; and (**B**) concentration-response curve for cardamonin on blocking the TRPA1 cation channel. Data are normalized to vehicle control and represent mean ± SEM, *n* = 3.

The concentration-dependent antagonistic effect of A-967079 was observed and the IC_50_ value was determined as 68.2 nM by this assay (Figure 2A), in accordance with previously reported results (67 nM by Ca^2+^ assay) [10]. In parallel, we assessed the potency of cardamonin on inhibiting TRPA1 activation at various concentrations. Interestingly, this was indeed the case. Full blockage of TRPA1 activity was achieved at 90 μM. The concentration-dependent inhibitory effect was observed and the IC_50_ value was defined as 454 nM (Figure 2B). These observations indicate that cardamonin holds strong inhibitory efficacy on the TRPA1 channel.

### 2.2. Selectivity Evaluation of Cardamonin on TRP Ion Channels

**Figure 3:**
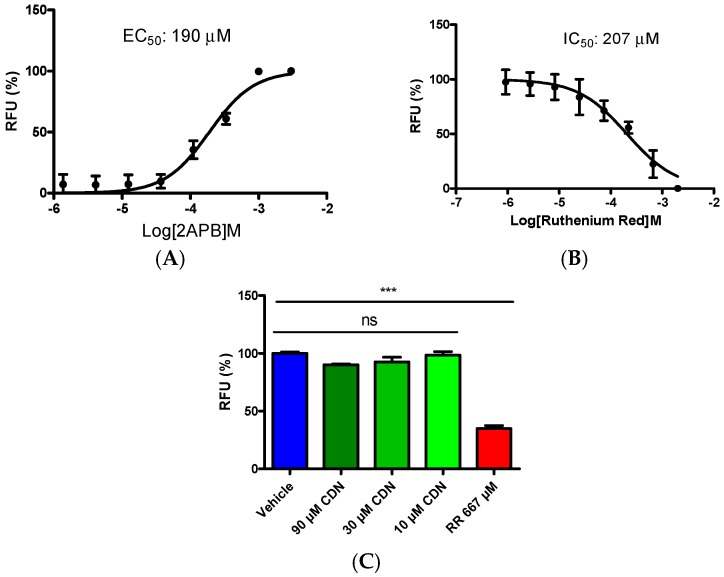
The effect of cardamonin on inhibiting TRPV1 activation. (**A**) Concentration-dependent activation of TRPV1 channel by 2APB; (**B**) inhibitory concentration-response curve for ruthenium red. The intracellular Ca^2+^ flux was assayed using FlexStation II plate reader the moment of adding 1 mM 2APB; and (**C**) statistical analysis of effects of cardamonin on TRPV1 activation. HEK293/TRPV1 cells were pre-treated with cardamonin for 10 min and then challenged with 1 mM 2APB at 37 °C. Data represent mean ± SEM, *n* = 3; ns, no significant differences, *** *p* < 0.001, by one-way ANOVA analysis.

TRPV1 and TRPA1 share high similarity in structure and function [20] and they can even form a complex [21]; thus, we wondered whether cardamonin inhibits the TRPV1 channel. A common activator, 2-Aminoethoxydiphenyl borate (2APB), was used to stimulate calcium mobilization in overexpressing HEK293/TRPV1 cells. A concentration-dependent agonistic response was induced by 2APB with an EC_50_ value determined as 190 μΜ (Figure 2A). With 1 mM 2APB stimulation, a concentration-dependent inhibitory response of ruthenium red was observed with IC_50_ value of 207 μΜ. Then, the effect of cardamonin was evaluated by being pre-incubated with HEK293/TRPV1 cells at 90 μΜ, 30 μΜ, and 10 μΜ, respectively. Calcium signals were captured simultaneously when adding 1 mM 2APB. The results showed that cardamonin failed to inhibit calcium influx in HEK293/TRPV1 cells (Figure 3C), suggesting that cardamonin could not block TRPV1 channel.

**Figure 4:**
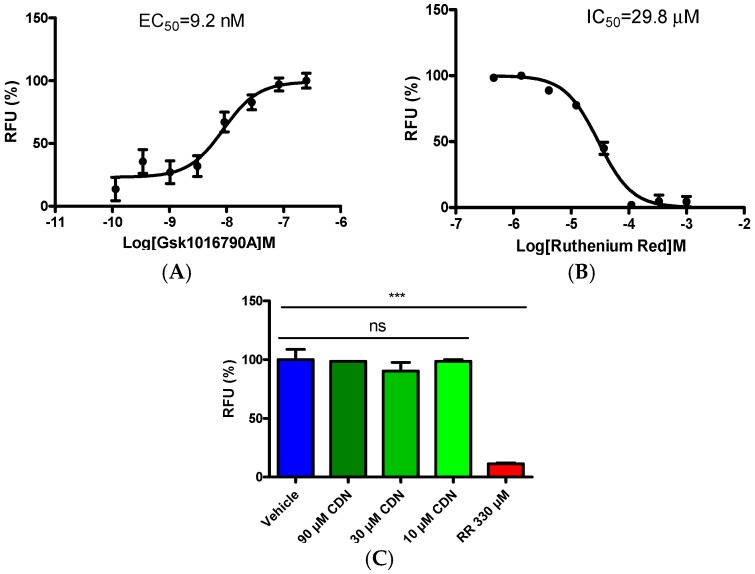
Assessment of cardamonin on TRPV4 activation. (**A**) Stimulating concentration-response curve of GSK1016790A on TRPV4 activation; (**B**) inhibitory concentration-response curve for ruthenium red (RR) on blocking TRPV4 activation. RR was added to the cells 10 min prior to 83 nM GSK1016790A stimulation. Calcium-flux measurement was conducted at 24 °C; and (**C**) statistical analysis of calcium signal in HEK293/TRPV4 cells treated with or without cardamonin. Data represent mean ± SEM, *n* = 3; ns, no significant differences, *** *p* < 0.001, by one-way ANOVA analysis.

TRPV4 is another critical TRP ion channel that plays a role in thermal and mechanical hyperalgesia [22,23]. TRPV4 shares similarity with TRPA1 [24,25]. To confirm whether cardamonin is a dual-inhibitor of TRPA1 and TRPV4, we further assessed its TRPV4-inhibitory potency in HEK293/TRPV4 cells with the specific agonist GSK1016790A. Firstly, the activating effect of the TRPV4 agonist GSK1016790A was tested. A concentration-dependent agonistic response was observed with an EC_50_ value of 9.2 nM (Figure 4A), in general accordance with previously reported results [26]. Then, the effect of a non-selective inhibitor ruthenium red on TRPV4-mediated currents was tested. The IC_50_ value was determined as 29.8 μΜ from the concentration-response curve (Figure 4B). Thereafter, cardamonin was incubated with HEK293/TRPV4 cells at 90 μM, 30 μM, and 10 μM prior to 83 nM GSK1016790A stimulation. HEK293/TRPV4 cells treated in the absence of the test compound was the negative control and 330 μM ruthenium red was performed as the positive control. No detectable inhibiting effect of cardamonin was observed (Figure 4C). This observation indicates that cardamonin does not interact with the TRPV4 ion channel.

### 2.3. Molecular Docking Analysis of Cardamonin with TRPA1 Protein

**Figure 5:**
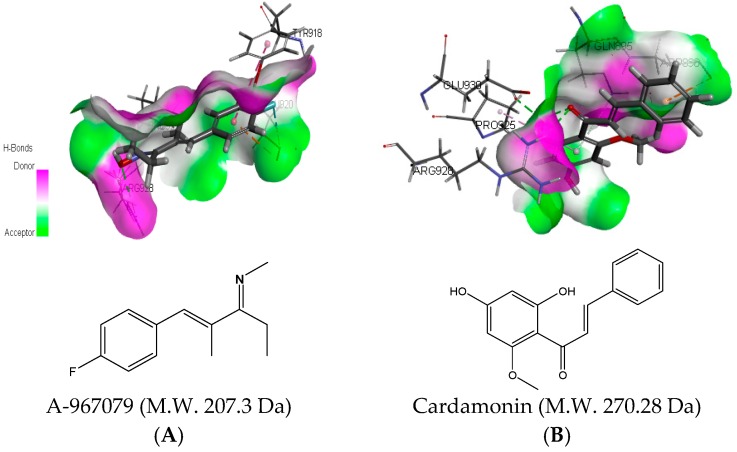
The optimal docking conformations of TRPA1 antagonists with the defined cavity in TRPA1 protein. (**A**) Representative hydrogen interaction of A-967079 with the active binding site; and (**B**) representative hydrogen interaction of cardamonin with key amino acids in the active binding cavity. The molecular docking poses were determined by Discovery Studio version 4.0 (Accelrys Software Inc., San Diego, CA, USA).

To further uncover the binding mechanism, we conducted molecular docking of cardamonin with TRPA1 protein. An active binding cavity was defined according three critical amino acids (Glu 920, Glu 924, and Glu 930) as described by Paulsen et al. [27]. The radius of the active binding site was 9.66. This active cavity was validated with the TRPA1 antagonist A-967079 with a K-score value of 62.23. Results showed that cardamonin aligned to TRPA1 through interaction with Arg 928, Pro 925, Glu 930, Gln 895, and Asp 896. The top K-score value of binding pose was detected as 71.82. In order to facilitate the analysis, the binding modes of A-967079 (Figure 5A) and cardamonin (Figure 5B) in the active pocket were superimposed. These observations confirms the activity of cardamonin with TRPA1 and reveals the potential putative binding information.

### 2.4. Cardamonins Scarcely Influenced HEK293 Cell Viability

**Figure 6:**
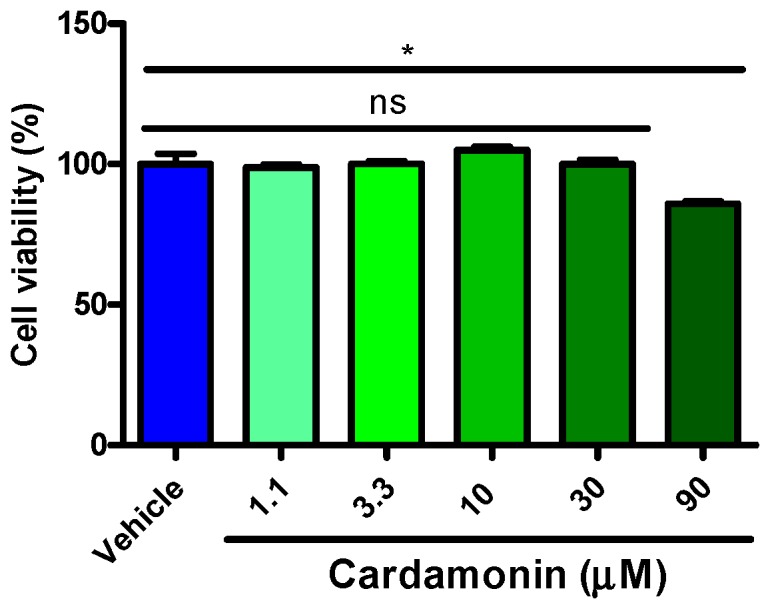
The effect of cardamonin on HEK293 cell viability. The cells were incubated with or without of cardamonin for 24 h. Cell viability was determined by Cell Titer-Glo assay. Data represent mean ± SEM, *n* = 3. ns, no significant differences, * *p* < 0.05, compared with vehicle control by one-way ANOVA analysis.

In order to rule out the possibilities of false positives, we tested whether cardamonin induced cytotoxicity in HEK293 cells. The cells were seeded into 96-well plate and incubated in the presence or absence of cardamonin. HEK293 cells were incubated with cardamonin for 24 h. Results indicated that cardamonin merely induced slight cell injury at a top concentration of 90 μΜ (Figure 6). However, no detectable cytotoxicity was induced by cardamonin within 30 μΜ (*p* > 0.05). This result indicates that cardamonin possesses a wide safety window between its pharmaceutical activity and cytotoxicity.

### 2.5. Cardamonin did not Inhibit Cardiomyocytes Contraction

**Figure 7:**
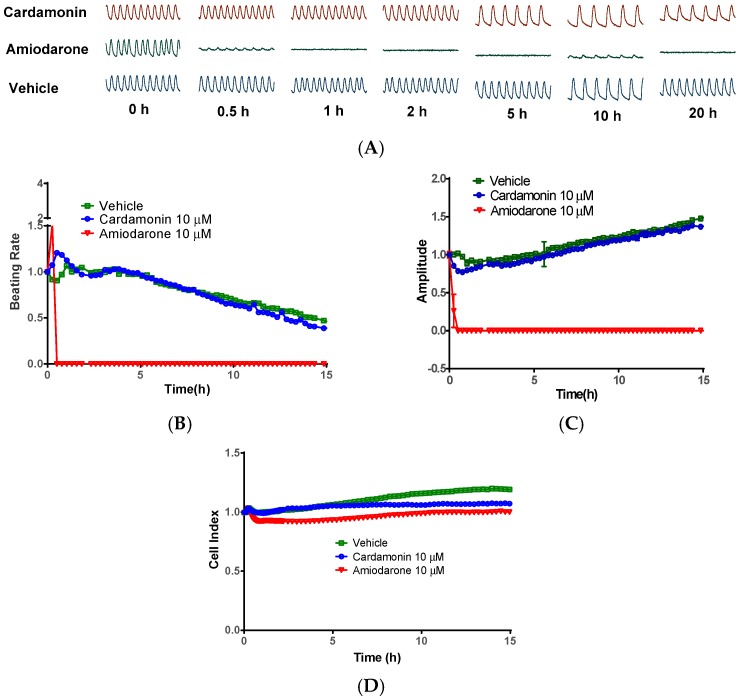
Cardiotoxicity evaluation of cardamonin on rat neonatal cardiomyocytes (CMs). (**A**) Contraction pattern of neonatal rat CMs in the presence or absence of cardamonin (10 μM); (**B**) real-time beating rate analysis of CM cells with test compound; (**C**) normalized amplitude analysis of CMs in the presence of cardamonin or amiodarone; and (**D**) real-time analysis of CMs’ cell index with cardamonin or amiodarone. The CMs cells incubated with vehicle was used as negative control. Data is a representative of three independent experiments.

TRPA1 is a non-selective calcium permeable ion channel, and blocking calcium influx in cardiomyocytes may impact cardio contraction [28]. The cation channel blockers may also disturb the cardiomyocytes’ beating activity [29]. Recent study revealed that TRPA1 ion channel was present in cardiac tissue [30]. To assess the potential adverse risk in cardiomyocytes, we administrated neonatal rat cardiomyocytes (CMs) with cardamonin, tested at 10 μM (22-fold of the IC_50_ value). Amiodarone (10 μM), an antiarrhythmic drug that delays cardiac repolarization through inhibiting hERG potassium channels [31], was performed as a positive cardio toxic control [32]. Real-time captured beating impedance showed that cardamonin did not induce dramatic alteration of CMs’ beating pattern (Figure 7A). Beating rate is a valuable phenotypic parameter for assessing cardiotoxicity. CMs’ beating rate was significantly decreased by amiodarone, but not by cardamonin treatment (Figure 7B). Amplitude represented the height of the print. The amplitude profile of cardamonin-treated CMs was in accordance with vehicle control (Figure 7C). Cell index represented cell number and viability. It could be observed that cardamonin did not significantly decrease CMs cell number and cell viability (Figure 7D). These observations imply that cardamonin is absent of clinical cardiotoxicity within its bioavailable doses.

## 3. Discussion

To the best of our knowledge, this is the first study that reports the antagonistic effect of cardamonin on the TRPA1 ion channel. Cardamonin is distinguished from the TRPA1 desensitization agonist nitro-oleic acid [33] since it could not activate TRPA1 alone. Our results demonstrate that cardamonin possesses no effect on anti-nociceptive ion channel TRPV1 or TRPV4, it could be deduced that cardamonin is a selective TRPA1 antagonist. The molecular docking analysis showed that cardamonin shared an A-967079-binding site, thus mutation of the critical amino acids revealed by Paulsen et al. [27] may fully abolish cardamonin antagonism and binding with TRPA1, while additional experiments are still warranted.

Activation of TRPA1 may up-regulate inflammatory factors and lead to pain. For example, in monosodium iodoacetate-induced joint pathology that mimicked osteoarthritis, enhanced interleukin-1 induced cyclooxygenase-2 (COX-2) expression. In contrast, the inflammatory effects were blunted by inhibition or depletion of TRPA1 [34]. TRP ion channels define the characteristic functional properties of nociceptors. TRPA1 antagonists target the very beginning of pain transduction, thus, are generally considered advantageous to traditional centrally acting drugs. TRPA1 antagonists exhibit less adverse responses such as sedation, dizziness, somnolence, or impairment of cognitive function [15]. These findings imply that cardamonin blocks the beginning of the pain pathway for transmission of nociception.

Cardiotoxicity was concerned with ion channel blockers; examples include hERG, Ca^2+^, and Na^+^ channel blockers [35]. As a TRPA1 antagonist, no obvious cardiotoxicity of cardamonin was observed towards cardiomyocytes. Absence of cardiotoxicity of another newly-identified TRPA1 antagonist (AMD_09) was also reported by Cristina et al. [36]. This indicates that cardiotoxicity is not typical for TRPA1 cation channel blockers.

In addition to inflammatory responses, a series of reports suggest that TRPA1 antagonists are useful to treat neuropathic pain, such as diabetic neuropathic pain and nerve injury induced pain. These activities were achieved through regulating neuropeptide calcitonin gene-related peptide (CGRP) release [37]. Antagonism of TRPA1 by A-967079 was reported to reduce free methylglyoxal and alleviate diabetic neuropathic pain [38]. Further, antagonism of TRPA1 could also relieve mechanical and cold allodynia that was stimulated by sciatic nerve chronic constriction injury [39]. This means that cardamonin may be able to attenuate neuropathic pain.

Our results also suggest potential applications of cardamonin for targeting cold pain pathways. TRPA1 is essential for cold induced vasoconstriction through controlling the CGRP release [40]. Cold exposure may deteriorate atherosclerotic plaque growth and improve lipolysis after TRPA1 activation, which indirectly increases the motility of heart stroke [41]. Studies have also shown that TRPA1 activation by cold stimulation markedly increased blood flow and this response was blocked by TRPA1 antagonists [42]. Therefore, antagonism of TRPA1 by cardamonin may offer possibilities for cold pain and cold induced vascular diseases [43], although further investigations are still demanded. Coincidently, *A. katsumadai* is a pungent and warm herbal medicine that holds potential for dispelling cold and wet. Blocking cold sensing TRPA1 ion channel with cardamonin, a constituent of *A. katsumadai*, may explain its ability to modulate thermal sensing.

## 4. Materials and Methods

### 4.1. Materials

Dulbecco’s modified Eagles’ medium (DMEM) and fetal bovine serum (FBS) were purchased from Gibco (Grand Island, NY, USA). Cardamonin and cinnamaldehyde were obtained from the National Institutes for Food and Drug Control (Beijing, China). GSK1016790A, Ruthenium Red, Amiodarone, A-967079, and 2APB were purchased from Sigma-Aldrich (Saint Louis, MO, USA). Other chemicals were also obtained from Sigma-Aldrich if not stated otherwise. Cell culture dishes and 96-well microplates were acquired from Greiner (Frickenhausen, Germany).

### 4.2. Calcium Mobilization Assay

Recombinant HEK293/TRPA1, HEK293/TRPV1, and HEK293/TRPV4 cell lines were generated by HD Bioscience Co. Ltd (Shanghai, China) following standard procedures as reported previously [44]. HEK293/TRPA1 and HEK293/TRPV4 cells were cultured in complete DMEM medium with 100 μg/mL hygromycin B, 50 U/mL penicillin, and 100 μg/mL streptomycin. HEK293/TRPV1 cells were cultured with 350 μg/mL zeocin and 5 μg/mL blasticidin in complete DMEM culture medium. 

Calcium influx upon TRPA1 activation by agonist stimulation was measured using calcium mobilization assay as reported previously [45]. Briefly, cells were plated into 96-well microplates (matrigel coated) at a density of 3.5 × 10^4^ cells per well. The plates were incubated at 37 °C with 5% CO_2_ overnight to allow the cells to recover. On the following day, replace cell culture medium with loading buffer containing Fluo-4 AM (MD, CA, USA), kept the plate at 37 °C for 30 min. Test compound was added to cells 10 min prior to the onset of TRAP1 agonist cinnamaldehyde, TRPV1 agonist 2APB, or TRPV4 agonist GSK1016790A. The calcium signal was captured with Flexstation II reader (MD, CA, USA) at 37 °C for TRPA1 and TRPV1 activation. The reader chamber was set at 24 °C for TRPV4 activation. 

### 4.3. Molecular Docking Calculations

Molecular docking was done using Discovery Studio™, version 4.0 (Accelrys Software Inc., San Diego, CA, USA). The X-ray crystal structure of TRPA1 ion channel (3J9P) was retrieved from Protein Data Bank [46]. Positive TRPA1 antagonist A-967079 and cardamonin were prepared with Discovery Studio and converted into three-dimensional (3D) structures. Energy minimizations of protein and small molecules were conducted by CHARMM force field. All solvent molecules within the protein structure were removed during protein preparation. The binding confirmation was done using the Libdock protocol. 

### 4.4. Cell Viability Analysis

HEK293 cell viability was determined based on quantification of ATP using Cell Titer-Glo^®^ luminescent Assay kit (Promega, Madison, WI, USA), which signals the presence of metabolically-active cells. Briefly, HEK293 cells were seeded into 96-well plates at a cell density of 3.5 × 10^4^ cells per well, the plates were kept at 37 °C with 5% CO_2_ overnight. On the following day, cardamonin was added to the cells from a top concentration of 90 μΜ. The cells treated in the absence of the test compound were the negative control. After incubated for 24 h, Cell Titer-Glo reagent was added to the cells and Luminescence was acquired on EnVision™ 2100 Multilabel Reader (PerkinElmer, Santa Clara, CA, USA).

### 4.5. Cardiotoxicity Evaluation

Cardiotoxicity test of cardamonin was performed with neonatal rat cardiomyocytes that obtained from 24 h Sprague-Dawley rats [47]. Compound treatment and cardiomyocytes impedance profiles were monitored as previously reported by Yu et al. [48]. Briefly, CMs cells were seeded into cardio E-Plate 96 that pre-coated with matrigel. Thereafter, cell culture medium was refreshed every day until CMs presented stable beating patterns. For compound treatment, 10× chemical dose solutions were prepared and added to the cells. The cell impedance was captured using xCELLigence RTCA cardio instrument.

### 4.6. Statistical Analysis

The data were representative of at least three independent experiments. Statistical significance of differences was assessed by one-way ANOVA using GraphPad Prism (GraphPad Software Inc., La Jolla, CA, USA). Statistically significant differences are indicated by *** *p* < 0.001, ** *p* < 0.01, and * *p* < 0.05.

## 5. Conclusions

Collectively, our data suggest that cardamonin is a selective TRPA1 antagonist, providing novel insight into its potency on inflammatory and neuropathic pain.
